# Exploring the variation in associations between socioeconomic indicators and non-communicable diseases in the Tromsø Study: an algorithmic approach

**DOI:** 10.1177/14034948241249519

**Published:** 2024-06-11

**Authors:** Sigbjørn Svalestuen, Emre Sari, Petja Lyn Langholz, Chi Quynh Vo

**Affiliations:** 1 Department of Social Sciences, UiT The Arctic University of Norway, Tromsø, Norway; 2 Health Services and Health Economics, NORCE Norwegian Research Centre AS, Tromsø, Norway; 3 Department of Archaeology, History, Religiuos Studies and Theology, UiT The Arctic University of Norway, Tromsø, Norway and; 4 Department of Community Medicine, UiT The Arctic University of Norway, Tromsø, Norway

**Keywords:** Non-communicable disease, socioeconomic status, machine learning, random forest, feature importance, prediction, partial dependence

## Abstract

**Aims:**

We contribute to the methodological literature on the assessment of health inequalities by applying an algorithmic approach to evaluate the capabilities of socioeconomic variables in predicting the prevalence of non-communicable diseases in a Norwegian health survey.

**Methods:**

We use data from the seventh survey of the population based Tromsø Study (2015–2016), including 11,074 women and 10,009 men aged 40 years and above. We apply the random forest algorithm to predict four non-communicable disease outcomes (heart attack, cancer, diabetes and stroke) based on information on a number of social root causes and health behaviours. We evaluate our results using the classification error, the mean decrease in accuracy, partial dependence statistics.

**Results:**

Results suggest that education, household income and occupation to a variable extent contribute to predicting non-communicable disease outcomes. Prediction misclassification ranges between 25.1% and 35.4% depending on the non-communicable diseases under study. Partial dependences reveal mostly expected health gradients, with some examples of complex functional relationships. Out-of-sample model validation shows that predictions translate to new data input.

**Conclusions:**

**Algorithmic modelling can provide additional empirical detail and metrics for evaluating heterogeneous inequalities in morbidity. The extent to which education, income and occupation contribute to predicting binary non-communicable disease outcomes depends on both non-communicable diseases and socioeconomic indicator. Partial dependences reveal that social gradients in non-communicable disease outcomes vary in shape between combinations of non-communicable disease outcome and socioeconomic status indicator. Misclassification rates highlight the extent of variation within socioeconomic groups, suggesting that future studies may improve predictive accuracy by exploring further subpopulation heterogeneity.**

## Introduction

Decades of studies rooted in traditional data modelling have produced convincing evidence for the existence and persistence of health inequalities. Particular attention has been given to the disease distribution between social groups and the social origins of disease [1–5]. However, issues of model dependence and the shortcomings of statistical significance as a single evaluation criterion [6, 7] suggest that existing metrics of scientific evaluation should be supplemented. Algorithmic modelling can provide such additional metrics by providing indicators of the predictive accuracy of the model in predicting new data input, and reduce model dependence through non-parametric estimation [8].

We therefore adopt an algorithmic modelling strategy to explore the variation in association between a set of socioeconomic status (SES) indicators and a set of non-communicable diseases (NCDs). We contribute to discussions on the statistical assessment of socioeconomic correlates of disease by applying an out-of-sample predictive approach in the context of a comprehensive Norwegian health survey. We apply the random forest [9] algorithm on classification problems, and compute variable importance statistics to assess to what extent these factors contribute to correctly predicting a history of NCD outcomes out of sample. We further compute partial dependences between SES indicators and NCD outcomes to assess how education, occupation and income aids the algorithm in making predictions. We discuss the potential benefits of integrating algorithmic modelling into the methodological toolbox of health inequality researchers.

## Methods and materials

### The Tromsø Study

The Tromsø Study is conducted in Tromsø municipality in northern Norway and aims to include large, representative samples of the local population. The invitation of whole birth cohorts and random sampling ensures a balanced representation of both genders, and a demographic closely mirroring the Tromsø population. The survey includes a wide range of variables, including both questionnaires, biological samples and clinical examinations. All inhabitants aged 40 years and older (*N*=32,591) were invited to Tromsø7 (2015–2016, 65% participated) [10].

Table I presents summary statistics on all predictors and outcomes applied in the algorithm. The overall sample size was *N=*21,083. We applied simple mean and median imputation to missing values. Our outcomes are four NCDs: diabetes, heart attack, stroke and cancer. These NCDs are leading causes of premature mortality and represent a great burden of disease globally [11]. The outcome measures comprise self-reports of both previous and current disease status. SES was measured using traditional indicators such as level of education, household income and occupational group. Following Olsen et al. [12] we categorised occupations as follows: unskilled (including semi-skilled manual jobs); intermediary (including office, sales, service and care jobs); lower professions requiring tertiary education of up to 3 years; and higher professions including administrative leaders, politicians, or professions requiring at least 4 years of tertiary education. Education was split into four categories: primary, vocational, tertiary education of less than 4 years and tertiary education of 4 years or more. Household income was measured as eight absolute income categories, ranging from less than NOK150,000 to greater than NOK1,000,000.

**Table I.** Summary statistics by sex and totals in the Seventh Tromsø Study (Tromsø7) 2015–2016.

Variables	Women		Men		Total		Variables	Women		Men		Total	
	*N*	Mean/%	*N*	Mean/%	*N*	Mean/%		*N*	Mean/%	*N*	Mean/%	*N*	Mean/%
Age	11,074	57.2	10,009	57.4	21,083	57.3	Consultation GP: Yes	9283	84.6	7569	76	16,852	80.5
BMI	11,038	26.9	9982	27.8	21,020	27.3	Consultation, specialist: Yes	2500	23.8	1682	17.4	4182	20.8
Education							Physical activity, leisure						
Primary/lower secondary	2617	24.1	2179	22.2	4796	23.2	Reading, watching TV etc.	1466	13.8	1506	15.4	2972	14.6
Vocational/upper secondary	2759	25.4	2997	30.5	5756	27.8	Walking, cycling etc. at least 4 h/week	6897	65	4918	50.4	11,815	58
College/university, <4 years	1917	17.6	2091	21.3	4008	19.4	Recreational sports, snow shoveling etc. at least 4 h/week	2000	18.8	2951	30.2	4951	24.3
College/university >4 years	3581	32.9	2564	26.1	6145	29.7	Hard training, several times/week	250	2.4	382	3.9	632	3.1
Household income (NOK thousands)							Employment						
<150	134	1.3	76	0.8	210	1.0	Works full time	5694	52.2	6354	64.6	12,048	58.1
150–250	635	6.1	355	3.6	990	4.9	Works part time	1248	11.4	414	4.2	1662	8
251–350	911	8.7	528	5.4	1439	7.1	Unemployed	53	0.5	84	0.9	137	0.7
351–450	1120	10.8	786	8.0	1906	9.4	Housekeeping	103	0.9	29	0.3	132	0.6
451–550	1319	12.7	993	10.2	2312	11.5	Retired	2526	23.1	2261	23	4787	23.1
551–750	1769	17.0	1803	18.5	3572	17.7	Student/military service	43	0.4	17	0.2	60	0.3
751–1000	2271	21.8	2470	25.3	4741	23.5	Disability benefit recipient/work assessment allowment	1239	11.4	662	6.7	1901	9.2
>1000	2257	21.7	2758	28.2	5015	24.8	Family income supplement	7	0.1	18	0.2	25	0.1
Occupation							Education, mother						
Unskilled	1567	14.6	3018	31.1	4585	22.5	Primary/lower secondary	7865	73.6	7075	72.9	14,940	73.3
Intermediary	4220	39.4	1716	17.7	5936	29.1	Vocational/upper secondary	1729	16.2	1671	17.2	3400	16.7
Lower profession	1486	13.9	1783	18.4	3269	16.0	College/university <4 years	697	6.5	659	6.8	1356	6.7
Higher profession	3429	32.0	3173	32.7	6602	32.4	College/university >4 years	400	3.7	294	3	694	3.4
NCD							Education, father						
Diabetes: Yes/yes, previously	527	4.9	597	6.1	1124	5.5	Primary/lower secondary	6350	59.9	5690	59.1	12,040	59.5
Heart attack: Yes/yes, previously	174	1.6	579	6.0	753	3.7	Vocational/upper secondary	2506	23.7	2400	24.9	4906	24.3
Cancer: Yes/yes, previously	868	8.1	768	7.9	1636	8.0	College/university <4 years	982	9.3	885	9.2	1867	9.2
Stroke: Yes/yes, previously	216	2.0	331	3.4	547	2.7	College/university >4 years	755	7.1	653	6.8	1408	7
Childhood economy							Economy, self-evaluated						
Very good	667	6.2	530	5.4	1197	5.8	Very good	1826	16.8	1800	18.3	3626	17.5
Good	7491	69.2	6659	68.2	14,150	68.7	Good	5468	50.3	5460	55.5	10,928	52.7
Difficult	2462	22.7	2410	24.7	4872	23.7	Average	3177	29.2	2249	22.9	5426	26.2
Very difficult	205	1.9	159	1.6	364	1.8	Difficult	360	3.3	258	2.6	618	3
Alcohol, frequency							Very difficult	50	0.5	69	0.7	119	0.6
Never	1089	9.9	604	6.1	1693	8.1	Occupation status, self-evaluated						
Monthly/less	3066	27.9	2073	20.8	5139	24.5	Very high social status	646	6	789	8.1	1435	7
2–4 Times/month	3954	36	3945	39.6	7899	37.7	Fairly high social status	3376	31.6	3784	38.8	7160	35
2–3 Times/week	2347	21.3	2628	26.4	4975	23.7	Neither high nor low status	5862	54.9	4591	47.1	10,453	51.1
4+/Week	537	4.9	709	7.1	1246	5.9	Fairly low status	735	6.9	535	5.5	1270	6.2
Smoke daily: Yes, now	1586	14.5	1318	13.3	2904	13.9	Very low status	66	0.6	57	0.6	123	0.6
Smoke daily: Yes, previously	4801	43.8	4449	44.8	9250	44.3	Live with spouse: Yes	7403	72.3	7880	81.6	15,283	76.8
Smoke daily: Never	4578	41.8	4155	41.9	8733	41.8							

Additional predictors were selected to include a broad range of social and demographic correlates of the socioeconomic indicators and health. We included key modifiable risk factors for NCDs such as alcohol consumption, physical activity level, body-mass index and smoking habits [13]. Indicators such as financial conditions in childhood and parental education level aim to capture the impact of early-life conditions on adult health outcomes [4]. We further included indicators of healthcare engagement (frequency of general practitioner and specialist consultations) as research has shown socioeconomic differences in general practitioner service provision [14], and inequalities in private medical specialist utilisation and hospital outpatient care [15]. Further, indicators of financial security and perceptions of occupational status aim to capture health effects related to relative social positioning and psychosocial stress [16]. Marital status was included to differentiate between single and dual household incomes. Finally, we include age and gender as demographic indicators.

### Ethical statement

All participants gave informed written consent, and the study was assessed by the Norwegian Center for Research Data (reference 869500). This study was not defined as health research by the Regional Ethics Committee North and was exempted from the requirement of study preapproval.

### Predicting in and out of sample

Stochastic models imply strong assumptions on the data generating process [17]. Approximating a true functional relationship with simple parametric models such as linear regression introduces some error. The difference between the estimated function and the empirical function is the model bias [18]. Predictive exercises for model validation are a common feature of this strategy in the form of goodness of fit tests and in-sample predictive power [6]. They tend to yield coefficient estimates that give the best in-sample predictions [8].

**Figure 1.**
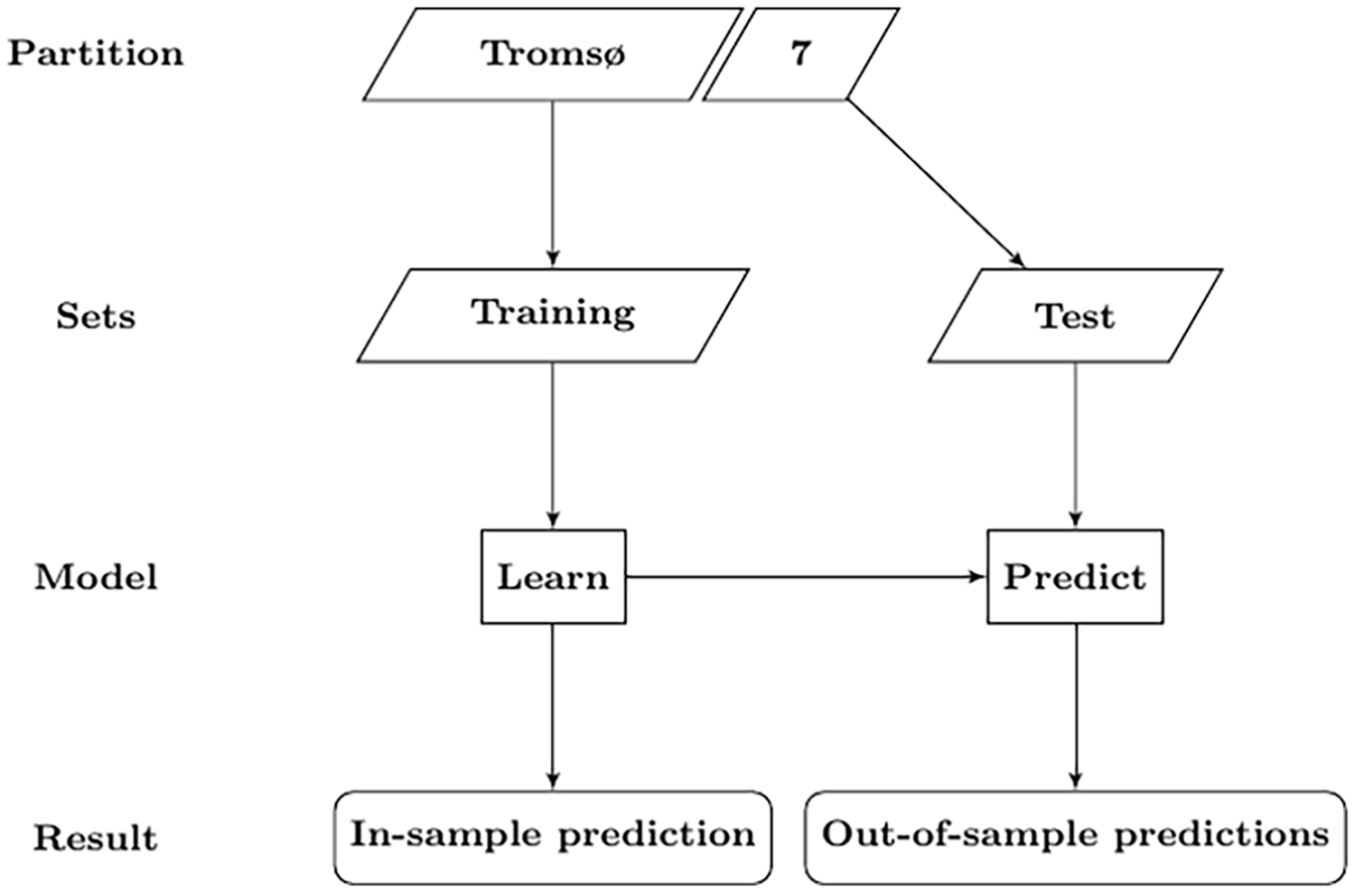
The train test methodology as applied in this paper. All steps are repeated when hyperparameters are tuned. All steps of the modelling procedure were performed for each outcome model.

Supervised machine learning methods search for functions that predict an output for the dependent variable given the independent variable input. Their goal is to achieve a balance between reducing the in-sample and out-of-sample errors, by searching for functions that are sufficiently complex to fit the data without fitting the underlying noise. Predictions are evaluated by their ability to forecast the outcome for future data inputs [8]. Figure 1 presents an overview of the train test methodology as applied in this paper. The goal of prediction is twofold; in-sample model fit evaluation, and out-of-sample model validation. For each model, the Tromsø7 sample is therefore partitioned into a training set and a test set. The models are produced on each training set and then applied to each test set. Models are validated by their generalisation error; the prediction error of a model when applied to a general population [6], as represented by the test set.

### The random forest algorithm

We approach this concept of prediction using the random forest algorithm. Random forest estimation grows many decision trees, allowing each tree to vote for the most popular class in classification problems [9]. A major advantage of the random forest approach is its ability to examine non-linear functional forms and complex interaction terms among covariates without the analyst having to prespecify a particular functional form or interaction term [19, 20], or the need for variable transformation [21]. We explore these patterns by first conducting a variable importance analysis by retrieving the estimated mean decrease in accuracy (MDA). MDA scores measure the mean decrease in classification performance when a given variable is excluded from the model [18] after permuting each element of the set of *Xj* predictors, in which *j i*ndexes each covariate, over all trees in the forest [19]. Second, we estimate partial dependences for education, household income and occupation. Partial dependence functions represent the effect of a given variable after accounting for the average effects of the other variables [22]. They represent the functional forms of the association between covariates and outcomes.

We apply the algorithm separately for each NCD. Outcomes are imbalanced, with non-NCD outcomes being much more common for all NCD outcomes. Class imbalances must be considered in classification models to avoid naive predictions. We achieve balanced outcomes by randomly selecting a subset of observations (with replacement) from both outcomes in the training set for each decision tree.

Each tree assumes that the population distribution of the given NCD outcome is equal to 50%, questioning the external validity of our sampling method. Therefore, before training the models and presenting the out-of-bag (OOB) error estimate, we hold out a random set equal to 20% of the data as a test set. Train test partitions are generated for each individual random forest model. Randomly partitioning the sets from the Tromsø7 dataset preserves the original data generating process, even when data are trained on a balanced subsample.

Random forests are estimated using the R package randomForest [23]. Partial dependences were calculated using the pdp package [24]. All models were estimated on 1000 decision trees.

## Results

### Prediction

Table II shows predictive accuracies from both training and test sets. The OOB error rate ranges from 25.6% for predicting a history of heart attack to 34% for the cancer outcome. False positives are more common than false negatives, except in diabetes predictions. The difference in classification error rate between classes is small for diabetes (–0.009), slightly higher for cancer (0.03), and the largest for heart attack (0.047) and stroke (–0.062). Comparing predictions from the test and training sets, we find only minor differences in performance between the OOB and test errors. Error rates for individual classes are also comparable to predictions based on the training set. Predictions from the training set assumed that group sizes were equal, as each tree was fit on a balanced class distribution by undersampling the majority class. Congruence between the test and OOB errors shows that the models translate to unbalanced out-of-sample class distributions, if the algorithm is trained on balanced data.

**Table II.** Prediction results from random forest training set and test set.

Training set						
NCD	OOB	False negative	False positive	Difference	Sample 0	Sample 1
Diabetes	0.306	0.306	0.315	–0.009	200	200
Stroke	0.289	0.287	0.349	–0.062	200	200
Heart attack	0.256	0.257	0.210	0.047	200	200
Cancer	0.340	0.343	0.312	0.030	200	200
Test set						
NCD	Test error	False negative	False positive	Difference	N (0)	N (1)
Diabetes	0.308	0.356	0.332	0.024	4001	216
Stroke	0.282	0.258	0.270	–0.012	4097	120
Heart attack	0.251	0.222	0.236	–0.014	4064	153
Cancer	0.354	0.328	0.341	–0.013	3897	320

NCD: non-communicable disease; OOB: out-of-bag.

### Variable importance and partial dependence

**Figure 2.**
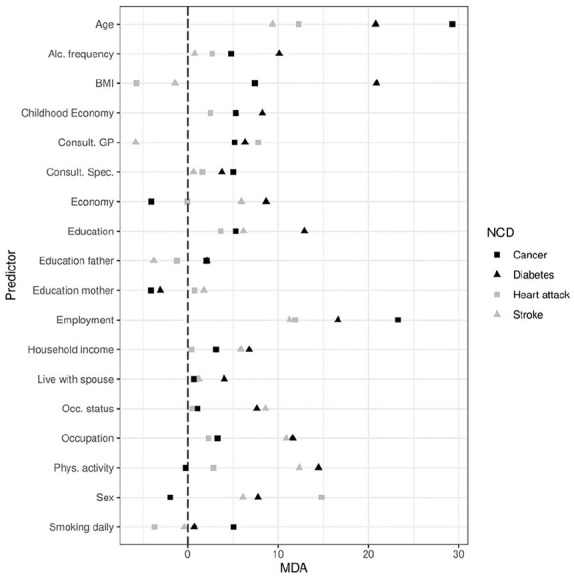
Variable importance (mean decrease in accuracy (MDA) for all predictors in the model, by non-communicable diseases (NCD).

Figure 2 presents variable importance in terms of the MDA score for all predictors over all outcomes. The MDA compares the differences between the error rate before and after permuting each predictor variable, normalising the score by dividing the average difference over all trees by their standard deviation [23]. Scores are therefore relative to their variance across all trees in the forest. While we concentrate our presentation on the socioeconomic predictors, variable importance scores are available for all predictors included in the model.

Education performs worst for predicting heart attack, contributes slightly more to predicting cancer and stroke, but is clearly the most effective at predicting diabetes out of the four NCDs. Household income increases the predictive accuracy for cancer, stroke and diabetes, but does not increase predictive accuracy for heart attack. This predictor contributes most to predicting diabetes and stroke, and shows some increase in accuracy in predicting cancer. Occupation increases predictive accuracy for diabetes and stroke outcomes, but seems less effective as a predictor for cancer and heart attack.

**Figure 3.**
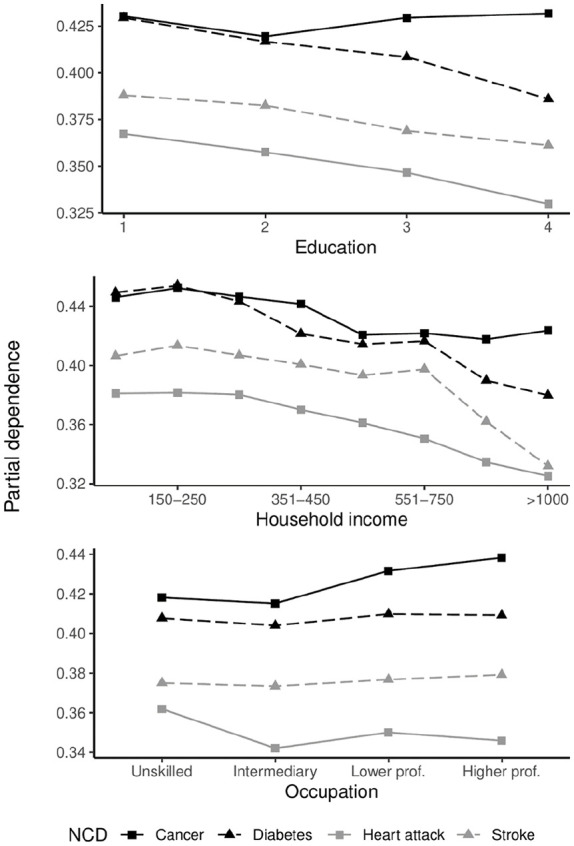
Partial dependence plot for three individual socioeconomic indicators. *Y*-axis represents the partial dependence; the proportion of trees voting for positive outcomes, averaged over all predictors in the model. *Y*-axis scaling is not standardised.

Partial dependence plots for education, household income and occupation are presented in Figure 3. Education gradients show decreases in the relative probability of positive classification for all NCD outcomes as education increases, except cancer. For the cancer outcome, the partial dependence increases post upper secondary education (category 2). For all other NCD outcomes, the education gradient shows minor deviation from a negative linear function.

Overall, partial dependence decreases as household income increases. For the cancer outcome, probabilities increase for those between the lowest and second lowest earners, starts dropping until reaching middle-income earners, showing a flatter but slightly positive gradient among high earners. Partial dependences between diabetes and income show a complex function that broadly separate low, middle and high-income groups, and show minor variations within these broader categories. The relationship between household income and stroke shows a gradient with a sharp decline towards the higher end of the income distribution. For the heart attack outcome, we observe a negative curvilinear reduction in partial dependence, with the effect tapering ever so slightly towards the top of the income distribution. While some minor differences can be found between occupational groups, there are no clear occupational gradients in diabetes and stroke based on prediction. There are greater differences between unskilled and other occupational groups for the heart attack outcome. The occupational gradient is largely similar to the educational gradient in cancer, likely due to some overlap in the definition of tertiary education and occupational categories 3 and 4.

## Discussion

We show that education, household income and occupation increase predictive accuracy for several NCDs. The empirical differences between groups are highlighted, as the partial dependences show that the probability of a given individual being classified with a positive outcome varies between most levels of education and income, and to a lesser extent occupation. The relationship between educational attainment, morbidity and mortality is empirically well established [1, ^4^]. For the predicted gradient between cancer and education, it is important to note that the outcome measure does not differentiate between cancer types. Certain types of cancer do show reverse SES gradients [1], and this is a likely driver of the results found in this study. However, the reverse gradient is small and defines a difference between participants with upper secondary education and those with tertiary education. Future predictive modelling studies should aim to distinguish different cancer outcomes when calculating gradients.

Household income predictions show a complex picture of NCD prevalence between reported income levels. The income-health gradient is long established, but the precise mechanisms remain up for debate [2, ^16^]. Results from this study show a complex function with several changes over the income range and substantial variation between the NCDs under study. While we cannot comment on the causal pathways between household income and NCD prevalence, the functional relationship presented by Figure 3 suggests that the issue is not restricted to issues of poverty, given the shape of the income gradients present in diabetes, stroke, heart attack, and to a lesser extent, cancer.

Occupational groups are associated with complex multimorbidities [5]. Occupation and education only partly explain the income-health gradient in Europe [25], suggesting that occupation provides separate causal pathways to health independent from income. A study using the same occupational indicator employed in this study identified an occupational gradient in self-rated health [12]. While we find that occupation improves prediction for diabetes and stroke, occupational gradients in these outcomes are comparatively flat from the perspective of the model. Occupational group differences may be statistically significant [5, ^12^], but lack predictive capabilities. An important finding regarding an occupation-health gradient is therefore that statistical significance does not imply accurate prediction, and that occupational gradients are sensitive to model and indicator selection. This is consistent with the general point highlighted in the literature [19] that statistical significance is neither necessary nor sufficient for predictive validity.

Elstad et al. [26] call for future studies on health inequalities in the Nordic countries to embrace causal inference techniques. Following examples in the broader methodological literature [6, 8, 27, 28], we argue that the methodological agenda should be expanded. Algorithmic approaches extend opportunities for causal inference via heterogeneous treatment effect estimation [8]. Further, these methodological paradigms overlap in key areas. For instance, matching techniques in causal inference and ensemble methods such as the random forest algorithm share similar goals in reducing model dependence.

### Strengths and limitations

Our approach has several strengths. The exploratory algorithmic approach to data analysis allows us to highlight the complex functions necessary for predicting a history of NCDs at the individual level. Our results further highlight the importance of indicator selection, and the non-parametric estimation procedure reduces issues that may arise from model dependence. This is important in the context of NCDs as their disease aetiology often defies the expectation of single cause explanations; rather, the risk of a given individual developing these diseases relies on many risk factors. Lundberg [29] argues that the communicative efficiency of paradigms such as the social determinants of health perspective suffers from determinism, and that social regularities cannot be translated into individual predictions because there are large individual variations within social groups. This is an important observation, but in its generalised state it risks missing the forest for the trees. Predictive metrics are necessary precisely because of subpopulation heterogeneity in the individual development of ill health and disease. They highlight not only the extent to which models make correct predictions; they equally highlight the cases in which predictions are wrong and, importantly, how incorrect predictions might occur (e.g. false positives/negatives). Presenting evidence from predictive metrics is therefore to a greater degree congruent with the disease aetiology of the NCDs under study than studies that emphasise statistical significance. Another strength of our study lies in the out-of-sample model validation. The relative congruence between the training and test set predictions shows that, if the data generating process within the Tromsø7 sample carries over to other data contexts, similar predictions are expected. A clear direction for future studies is therefore to evaluate empirically the extent to which the data generating process in the Tromsø study indeed translates to external data contexts. Our study provides a baseline for which future studies may refine their predictive efforts in the context of health inequalities in NCD prevalence.

Our approach is, however, not without limitations. Simultaneity issues are present in all cross-sectional studies. Interpreting variable importance scores for specific features thus requires some caution. Due to the retrospective and cross-sectional nature of the data, we cannot disentangle those who change their behaviours after receiving a diagnosis from those who do not. This may negatively impact the predictive power of factors such as smoking, alcohol consumption and physical activity. Those individuals with a high healthcare uptake will include those that visit their general practitioner and seek specialist care because of their illness, possibly inflating their predictive importance. The large MDA feature importance score observed for employment status may in part reflect the impact of ill health and health shocks on the probability of employment and labour market exit identified in the literature [30]. Despite these limitations, the MDA scores for education, income and occupational group show that predictive models including socioeconomic indicators will outperform predictive models in which socioeconomic indicators are absent; even in a predictor space including proximal risk factors and indicators sensitive to simultaneity issues. Congruence in predictions between the training set and the test set suggests that overfitting is not a substantial issue when these predictors are included. Further, the partial dependence metrics show that the algorithm uses the information in the socioeconomic indicators to create socioeconomic gradients in health when averaged over all other predictors in the model.

## Conclusions

Results from algorithmic modelling show that the extent to which socioeconomic status contributes to predicting binary NCD outcomes depends on the NCD and the choice of socioeconomic indicator. Evaluating partial dependences reveals that social gradients in NCD outcomes vary in shape between combinations of NCD outcome and socioeconomic indicator. Misclassification rates highlight the extent of variation within socioeconomic groups, suggesting that future studies may improve predictive accuracy by exploring further subpopulation heterogeneity.

There is ample opportunity to leverage predictive modelling further in Norway, due to the vast amount of population data stored in central registries and large cohort surveys. Future studies should apply predictive algorithms in a longitudinal context, such that information on changes in individual behaviour and timing of disease onset can be exploited in investigations on the predictive contributions that socioeconomic status makes.
